# The impact of heart failure and chronic obstructive pulmonary disease on mortality in patients presenting with breathlessness

**DOI:** 10.1007/s00392-018-1342-z

**Published:** 2018-08-08

**Authors:** Joseph J. Cuthbert, Joshua W. Kearsley, Syed Kazmi, Anna Kallvikbakka-Bennett, Joan Weston, Julie Davis, Stella Rimmer, Andrew L. Clark

**Affiliations:** Department of Academic Cardiology, Hull York Medical School, Hull and East Yorkshire Medical Research and Teaching Centre, Castle Hill Hospital, Cottingham, Kingston upon Hull, HU16 5JQ UK

**Keywords:** Heart failure, Chronic obstructive pulmonary disease, COPD, Mortality, Outcome, Prognosis

## Abstract

**Background:**

Differentiating heart failure from chronic obstructive pulmonary disease (COPD) in a patient presenting with breathlessness is difficult but may have implications for outcome. We investigated the prognostic impact of diagnoses of COPD and/or heart failure in consecutive patients presenting to a secondary care clinic with breathlessness.

**Methods:**

In patients with left ventricular systolic dysfunction (LVSD) by visual estimation, N-terminal pro B-type natriuretic peptide (NTproBNP) levels and spirometry were evaluated (*N* = 4986). Heart failure was defined as either LVSD worse than mild (heart failure with reduced ejection fraction) or LVSD mild or better and raised NTproBNP levels (> 400 ng/L) (heart failure with normal ejection fraction). COPD was defined as forced expiratory volume in 1 s (FEV_1_) to forced vital capacity (FVC) ratio < 0.7. The primary outcome was all-cause mortality.

**Results:**

1764 (35%) patients had heart failure alone, 585 (12%) had COPD alone, 1751 (35%) had heart failure and COPD, and 886 (18%) had neither. Compared to patients with neither diagnosis, those with COPD alone [hazard ratio (HR) = 1.84 95% confidence interval (CI) 1.40–2.43], heart failure alone [HR = 4.40 (95% CI 3.54–5.46)] or heart failure and COPD [HR = 5.44 (95% CI 4.39–6.75)] had a greater risk of death. COPD was not associated with increased risk of death in patients with heart failure on a multivariable analysis.

**Conclusion:**

While COPD is associated with increased risk of death compared to patients with neither heart failure nor COPD, it has a negligible impact on prognosis amongst patients with heart failure.

**Electronic supplementary material:**

The online version of this article (10.1007/s00392-018-1342-z) contains supplementary material, which is available to authorized users.

## Introduction

Breathlessness is common. Separating heart failure from chronic obstructive airways disease (COPD), both or neither can be difficult due to similarity in symptomatology and shared risk factors, such as increasing age and smoking history. However, the distinction is important: there are numerous drug and device therapies that can prolong life in patients with heart failure and reduced ejection fraction (HeFREF) [1], but there is little evidence that commonly used treatments of COPD affect mortality [2]. In addition, patients with heart failure who are also diagnosed with COPD often receive suboptimal treatment, as they are less likely to be prescribed beta-blockers due to concerns about bronchoconstriction [3].

The ratio of forced expiratory volume in 1 s (FEV_1_) to forced vital capacity (FVC) below 0.7 indicates obstructive pulmonary disease [4], and many patients with heart failure also have obstructive spirometry [5, 6]. Estimates of the prevalence of COPD amongst patients with heart failure vary between 8 and 52% depending on the definition of COPD [7, 8], but the impact of a co-diagnosis of COPD and heart failure is not clear.

The European Society of Cardiology heart failure guidelines state that the presence of COPD (regardless of the definition) is associated with worse prognosis in patients with HeFREF. However, numerous studies investigating the effect of COPD on outcome in patients with heart failure have been inconsistent [6, 9–22]. Studies that have reported increased mortality amongst patients with heart failure and COPD compared with either diagnosis alone have either come from highly selected trial populations of patients with HeFREF [9, 15, 19], patients admitted with acute heart failure [6, 11, 16], or have involved only short-term follow-up [10, 12, 20]. Up to half of patients with heart failure have a normal ejection fraction (HeFNEF) [23], but the prognostic significance of COPD amongst outpatients with HeFNEF has, so far, only been assessed in a small number of patients [22].

Reliable data on the long-term impact of a diagnosis of COPD on outcome in “real-world” outpatients with heart failure is scarce. We therefore assessed the impact of a diagnosis of COPD and/or heart failure on long-term mortality in a large cohort of consecutive ambulatory patients with breathlessness referred to a secondary care clinic with suspected heart failure.

## Methods

### Setting, study design and patients

Between September 2000 and October 2016, all clinical, demographic, biochemical and echocardiographic data on consecutive patients referred from primary or secondary care to a community heart failure clinic serving a local population of about 500,000 people were recorded on a secure database (Hull LifeLab). Patients were followed-up until 1st November 2016. All subjects gave their written informed consent for their data to be used. The study conforms to the principles outlined in the Declaration of Helsinki and was approved by relevant ethical bodies.

### Definitions and outcome

Heart failure was defined as the presence of signs and symptoms of the disease and either left ventricular dysfunction (LVSD) worse than mild—HeFREF; or LVSD mild or better and N-terminal peptide of pro B-type natriuretic peptide (NTproBNP) level > 400 ng/L—HeFNEF [24]. Spirometry was performed routinely on all patients at baseline by trained nursing staff. COPD was defined as FEV_1_:FVC < 0.7 as suggested by the GOLD criteria [4]. The primary outcome was all-cause mortality.

### Statistical analysis

Categorical data are presented as number and percentages, normally distributed continuous data are presented as mean ± standard deviation (SD) and non-normally distributed variables are presented as median and interquartile range (IQR).

The relationship between FEV_1_:FVC and other variables was assessed by Pearson’s correlation coefficient and linear regression. Variables associated with COPD as a categorical variable (FEV_1_:FVC < 0.7) were assessed using logistic regression. Only variables with *P* < 0.1 (an arbitrary threshold) in univariable analysis were included in multivariable models. Log-transformed NTproBNP was used.

Patients with were divided into groups: heart failure alone; COPD alone; heart failure and COPD; and neither diagnosis. Patients with heart failure were sub-divided into those with HeFREF or HeFNEF. Chi-squared tests were used to compare categorical variables and one-way analysis of variance (ANOVA) to compare continuous variables across the groups. Assumptions of ANOVA (normality of residuals and equal variance) were checked. Kruskal–Wallis test was used to compare non-normally distributed continuous variables across quartiles and groups.

Associations between variables and outcome were assessed with Cox regression. Proportionality of hazards was checked by residual plotting. Univariable analysis was conducted using all variables in the dataset: variables with *P* > 0.1 in univariable analysis or with more than 10% missing values (an arbitrary threshold) were not included in the multivariable analyses. Kaplan–Meier curves were used to demonstrate outcome by group.

All statistical analyses were carried out using the SPSS 24 software package with the two-tailed level of statistical significance set at *P* < 0.05.

## Results

Of 4986 patients, 1764 (35%) had heart failure alone, 585 (12%) had COPD alone, 1751 (35%) had both heart failure and COPD, and 886 (18%) had neither condition (Table 1). Of the 3515 total patients with heart failure, 2329 had HeFREF and 1186 had HeFNEF. The prevalence of COPD reported in the medical record (10% for HeFREF; 10% HeFNEF) was far lower than the prevalence of COPD by spirometry (49% for HeFREF; 51% for HeFNEF), but was similar between the groups (*P* = 0.20) (Table 2) (Supplementary Fig. 1).

**Table 1:** Patient characteristics: all patients

Variable	Missing	All patients	Heart failure	COPD	Heart failure and COPD	Neither heart failure nor COPD	*P*
		*N* = 4986	*N* = 1764	*N* = 586	*N* = 1750	*N* = 886	
Demographics							
Age (years)	0	71 (± 11)	71 (± 12)	71 (± 10)	75 (± 10)	66 (± 12)	< 0.001
Sex (male)—*N* (%)	0	3068 (62)	1197 (68)	273 (47)	1139 (65)	459 (52)	< 0.001
BMI (kg/m^2^)	17	28.7 (± 6.0)	29.1 (± 6.1)	29.2 (± 5.6)	27.4 (± 5.7)	30.1 (± 6.2)	< 0.001
SR [*N* (%)]	80	3562 (71)	1087 (63)	559 (96)	1060 (62)	3562 (73)	< 0.001
Diabetes [*N* (%)]	308	970 (20)	407 (24)	80 (15)	334 (21)	149 (18)	< 0.001
IHD [*N* (%)]	31	2259 (45)	912 (52)	151 (26)	935 (54)	261 (30)	< 0.001
Documented COPD in medical records [*N* (%)]	31	478 (10)	118 (7)	86 (15)	223 (13)	51 (6)	< 0.001
Never smoked [*N* (%)]	319	1421 (30)	536 (33)	158 (29)	383 (24)	344 (41)	< 0.001
Symptoms							
NYHA Class III/IV [N (%)]	245	1344 (28)	537 (31)	90 (17)	617 (36)	100 (14)	< 0.001
Blood results							
NTproBNP [ng/L]	461	723 (179–2045)	1319 (656–2821)	140 (73–228)	1590 (742–3309)	113 (57–203)	–
Haemoglobin [g/dL]	395	13.5 (12.3–14.5)	13.4 (12.1–14.5)	13.8 (12.9–14.7)	13.2 (12.0–14.3)	13.9 (13.0–14.9)	< 0.001
eGFR [mL/min/1.73 m^2^]	346	62 (48–77)	59 (44–74)	71 (58–83)	56 (42–70)	74 (62–87)	< 0.001
Albumin [g/L]	341	38 (36–40)	38 (35–40)	39 (37–40)	37 (35–39)	39 (37–41)	< 0.001
Spirometry							
FEV_1_ [L]	0	1.76 (± 0.81)	1.96 (± 0.76)	1.51 (± 0.72)	1.38 (± 0.60)	2.30 (± 0.87)	< 0.001
FVC [L]	0	2.58 (± 1.00)	2.46 (± 0.95)	2.78 (± 1.07)	2.49 (± 0.95)	2.87 (± 1.09)	< 0.001
FEV_1_:FVC	0	0.69 (± 0.16)	0.80 (0.09)	0.54 (± 0.13)	0.56 (± 0.12)	0.80 (± 0.09)	–
Medications							
Loop diuretic [*N* (%)]	107	2932 (60)	1240 (71)	191 (34)	1265 (73)	236 (28)	< 0.001
MRA [*N* (%)]	107	886 (18)	443 (25)	25 (5)	382 (22)	36 (4)	< 0.001
ACEI or ARB [*N* (%)]	107	3142 (63)	1266 (73)	248 (44)	1250 (72)	378 (45)	< 0.001
βB [*N* (%)]	107	2503 (50)	1090 (63)	170 (30)	948 (55)	295 (35)	< 0.001
Echocardiography							
Severe LVSD [*N* (%)]	0	411 (8)	207 (12)	0 (0)	204 (12)	0 (0)	–
HeFNEF [*N* (%)]	0	1186 (24)	583 (33)	0 (0)	603 (34)	0 (0)	–

Continuous data are presented as mean (± standard deviation) or median (interquartile range), categorical data are presented as *N* (percentage)

*N* number; *COPD* chronic obstructive pulmonary disease; *BMI* body mass index; *SR* sinus rhythm; *IHD* ischaemic heart disease; *NYHA* New York Heart Association; *NTproBNP* N-terminal B-type natriuretic peptide; *eGFR* estimated glomerular filtration rate; *FEV*_*1*_ forced expiratory volume in one second; *FVC* forced vital capacity; *MRA* mineralocorticoid receptor antagonist; *ACEI* angiotensin converting enzyme inhibitor; *ARB* angiotensin receptor blocker; *βB* beta-blocker; *LVSD* left ventricular systolic dysfunction; *HeFNEF* heart failure with normal ejection fraction

**Table 2:** Patient characteristics: all patients with heart failure (*N* = 3515)

Variable	Missing	All patients with heart failure	HeFREF	HeFNEF	HeFREF and COPD	HeFNEF and COPD	*P*
		*N* = *3515*	*N* = *1181*	*N* = *583*	*N* = *1148*	*N* = *603*	
Demographics							
Age (years)	0	73 (± 11)	69 (± 12)	75 (± 10)	73 (± 10)	77 (± 8)	< 0.001
Sex (male)—*N* (%)	0	2336 (67)	871 (74)	326 (56)	851 (74)	288 (48)	< 0.001
BMI [kg/m^2^]	13	28.3 (± 5.9)	28.7 (± 5.7)	29.9 (± 6.8)	26.9 (± 5.2)	28.4 (± 6.3)	< 0.001
SR [*N* (%)]	65	2148 (62)	809 (70)	278 (48)	754 (67)	307 (52)	< 0.001
Diabetes [*N* (%)]	206	742 (22)	278 (25)	129 (24)	216 (20)	119 (21)	0.07
IHD [*N* (%)]	12	1848 (53)	713 (61)	199 (35)	735 (64)	201 (33)	< 0.001
Documented COPD in medical records [*N* (%)]	12	341 (10)	74 (6)	44 (8)	149 (13)	74 (12)	< 0.001
Never smoked [*N* (%)]	237	919 (28)	332 (30)	204 (37)	215 (20)	168 (30)	< 0.001
Symptoms							
NYHA Class III/IV [*N* (%)]	59	1154 (33)	373 (33)	164 (28)	424 (38)	193 (33)	< 0.001
Blood results							
NTproBNP (ng/L)	461	1421 (695–3109)	1444 (536–3486)	1192 (737–2241)	1825 (708–4013)	1328 (773–2542)	< 0.001
Haemoglobin (g/dL)	294	13.3 (12.1–14.4)	13.6 (12.3–14.7)	13.1 (11.8–14.2)	13.3 (12.2–14.4)	12.9 (11.7–14.2)	< 0.001
eGFR (mL/min/1.73 m^2^)	287	58 (43–72)	59 (46–74)	59 (43–74)	56 (43–70)	56 (41–69)	< 0.001
Albumin (g/L)	281	37 (35–40)	38 (35–40)	37 (35–39)	37 (35–40)	37 (34–39)	< 0.001
Spirometry							
FEV_1_:FVC	0	0.68 (± 0.16)	0.80 (± 0.10)	0.80 (± 0.08)	0.56 (± 0.12)	0.56 (± 0.11)	< 0.001
Medications							
Loop diuretic [*N* (%)]	37	2505 (72)	872 (75)	368 (64)	857 (75)	408 (68)	< 0.001
MRA [*N* (%)]	37	825 (24)	371 (32)	72 (13)	309 (27)	73 (27)	< 0.001
ACEI or ARB [*N* (%)]	37	2517 (72)	906 (78)	360 (62)	890 (78)	361 (61)	< 0.001
βB [*N* (%)]	37	2039 (59)	744 (64)	346 (60)	639 (56)	310 (52)	< 0.001
Echocardiography							
Severe LVSD [*N* (%)]	0	916 (26)	457 (39)	0 (0)	459 (40)	0 (0)	–
LVEF by Simpsons (%)	1511	38.2 (± 12.6)	32.8 (± 8.0)	54.6 (± 6.7)	32.4 (± 8.2)	55.8 (± 7.64)	–

Continuous data are presented as mean (± standard deviation) or median (interquartile range), categorical data are presented as *N* (percentage)

*N* number; *HeFREF* heart failure with a reduced ejection fraction; *HeFNEF* heart failure with normal ejection fraction; *COPD* chronic obstructive pulmonary disease; *BMI* body mass index; *SR* sinus rhythm; *IHD* ischaemic heart disease; *NYHA* New York Heart Association; *NTproBNP* N-terminal B-type natriuretic peptide; *eGFR* estimated glomerular filtration rate; *FEV*_*1*_:*FVC* ratio of forced expiratory volume in one second to forced vital capacity; *MRA* mineralocorticoid receptor antagonist; *ACEI* angiotensin converting enzyme inhibitor; *ARB* angiotensin receptor blocker; *βB* beta-blocker; *LVSD* left ventricular systolic dysfunction; *LVEF* left ventricular ejection fraction

### Patient characteristics

Patients with COPD alone had similar NTproBNP levels and symptom severity but were older than patients with neither heart failure nor COPD. Atrial fibrillation was far less common in patients with COPD alone than in those with heart failure alone, heart failure and COPD or neither condition.

Patients with heart failure alone were similar in their age and smoking history but had more severe symptoms than patients with COPD alone. Patients with heart failure and COPD had similar symptom severity and rate of loop diuretic prescription but were older, had higher NTproBNP levels, and were less likely to be prescribed a beta-blocker than patients with heart failure alone.

Patients with heart failure regardless of the presence of COPD were more likely to be male, have ischaemic heart disease and had a lower estimated glomerular filtration rate (eGFR) than patients without heart failure (Table 1).

Compared to patients with HeFREF, patients with HeFNEF were older, more likely to be female, less likely to have ischaemic heart disease (IHD), less likely to have severe symptoms, less likely to be prescribed loop diuretic and had lower NTproBNP levels (Table 2).

### Associations with FEV_1_:FVC < 0.7

On univariable analysis, there were weak positive correlations between FEV_1_:FVC ratio and body mass index (BMI), haemoglobin, eGFR and albumin; there were weak negative correlations between FEV_1_:FVC ratio and age and NTproBNP (Supplementary Fig. 2). Of these, only age (β coefficient = − 0.14; *t*-statistic = − 7.8) and BMI (*β* = 0.10; *t* = 6.4) correlated with FEV_1_:FVC on multivariable analysis (data not shown).

### Outcome for all patients

During a median follow-up of 1825 days (IQR 802–1825), 1551 patients died. Decreasing FEV_1_:FVC as a continuous variable and COPD as a categorical variable were associated with an increased risk of mortality in all patients, but not after adjustment for other variables such as age, NTproBNP, eGFR and NYHA class (Supplementary Table 1).

**Fig. 1:**
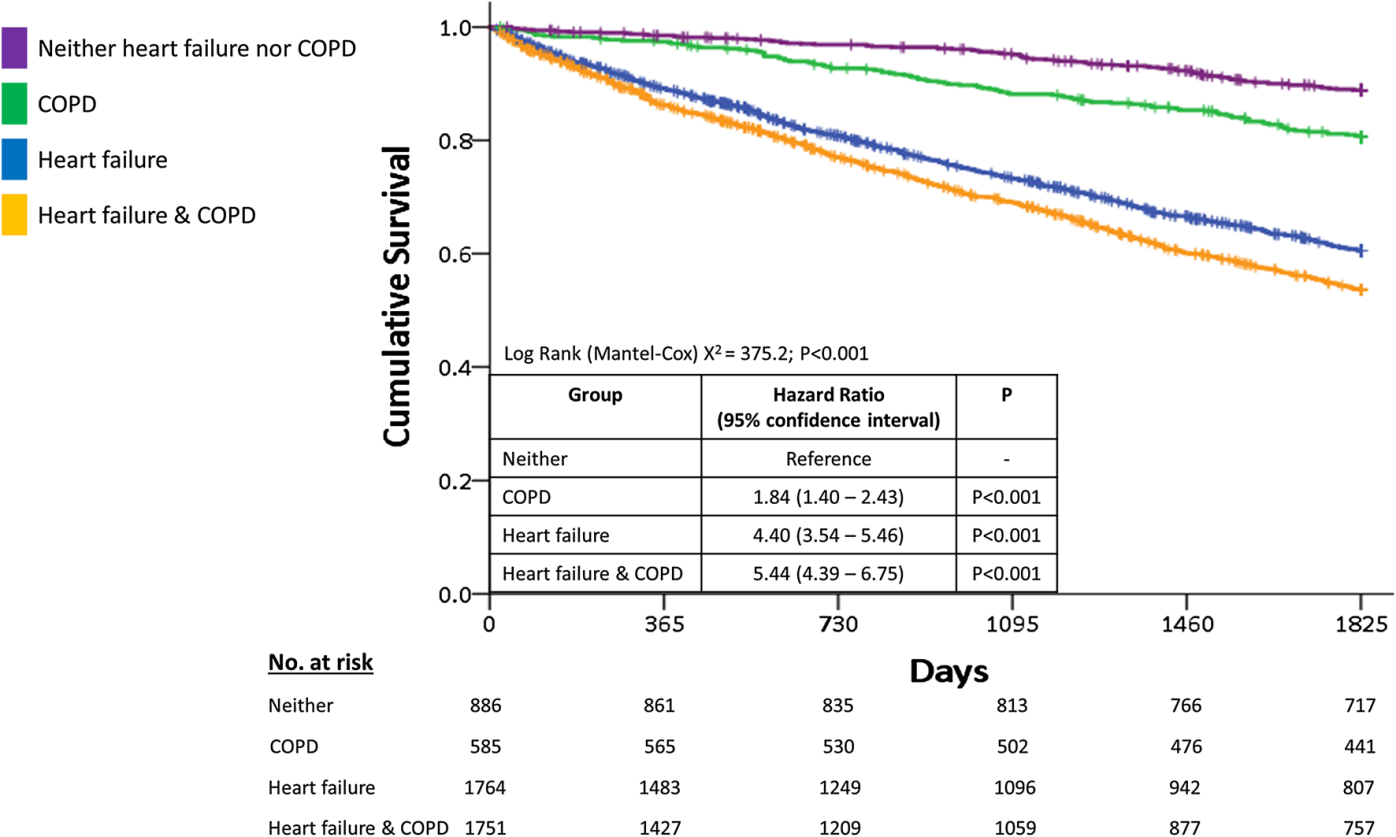
Survival curves for patients with heart failure, COPD, both or neither. Kaplan–Meier curves for 5-year mortality for patients with heart failure, COPD, both or neither. Abbreviations used: *COPD* chronic obstructive pulmonary disease. *No* number

Patients with COPD alone were nearly twice as likely to die as patients with neither condition and patients with heart failure regardless of the presence of COPD were 4–5 times more likely to die than patients with neither condition (Fig. 1).

### Outcome for patients with heart failure

**Fig. 2:**
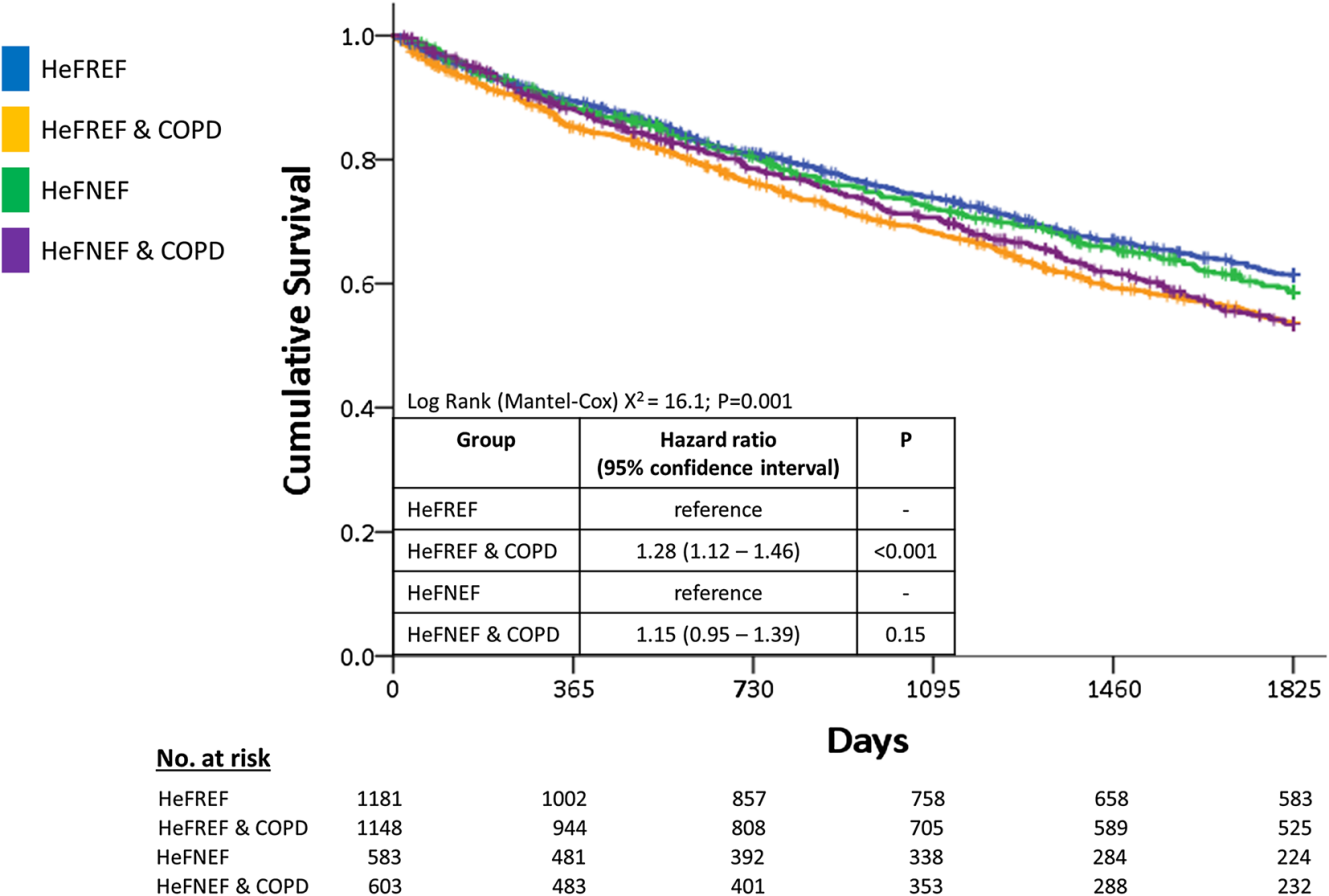
Survival curves for patients with either HeFREF or HeFNEF with or without COPD. Kaplan–Meier curves for 5-year mortality for patients with heart failure with or without COPD. Abbreviations used: *COPD* chronic obstructive pulmonary disease; *HeFNEF* heart failure with normal ejection fraction; *HeFREF* heart failure with reduced ejection fraction; *No* number

Amongst all patients with heart failure, during a median follow-up of 1534 days (IQR 572–1825), 1347 patients died. Decreasing FEV_1_:FVC as a continuous variable and COPD as a categorical variable were associated with poor prognosis on univariable analysis. The presence of COPD was associated with poor prognosis on univariable analysis amongst patients with HeFREF but not amongst patients with HeFNEF (Fig. 2). Neither FEV_1_:FVC as a continuous variable nor COPD as a categorical variable were associated with outcome after adjustment for other variables (Supplementary Table 2).

## Discussion

We have found that approximately half of patients with heart failure seen in a secondary care clinic have COPD (defined as FEV_1_:FVC < 0.7). Compared to patients with neither heart failure nor COPD, those with COPD alone were at a higher risk of death, but those with heart failure had a far greater risk of mortality regardless of the presence of COPD.

### The effect of a COPD diagnosis on heart failure

The diagnosis of COPD in a patient with heart failure (regardless of phenotype) has a negligible impact on outcome. This runs counter to the assertion in the current European Society of Cardiology heart failure guidelines that, regardless of definition, COPD is associated with worse outcome in patients with HeFREF [1].

Of all the studies that have demonstrated a significant association between the presence of COPD and increased risk of adverse outcome in patients with heart failure [6, 9–12, 15, 16, 18–20, 22] only three defined COPD using spirometry and heart failure using echocardiography or natriuretic peptide levels [6, 20, 22]; of these three studies, only one adjusted for natriuretic peptide levels and found an association between COPD and all-cause mortality or cardiovascular hospitalisation, but in a study of only 71 patients with HeFNEF [22]. Natriuretic peptide levels are powerful prognostic markers in patients with heart failure and may also be useful prognostic biomarkers in patients with COPD [25, 26]. Our results suggest that in patients with heart failure, it is high natriuretic peptide levels (a marker of heart failure severity), not the presence of COPD, which increases the risk of adverse outcome.

However, the diagnosis of COPD in a patient with heart failure may have important implications for management. We found that the prevalence of COPD identified by abnormal spirometry is higher amongst patients with heart failure than recorded in the medical notes: COPD may be missed when breathlessness is attributed to the heart failure diagnosis. Registry data suggest that most patients with heart failure and COPD have the latter diagnosis confirmed without spirometry [27]. Indeed, most studies claiming to investigate the impact of COPD on outcome in patients with heart failure do not use spirometry to define COPD [9–11, 14–16, 19]. Under-diagnosis of COPD amongst patients with heart failure may be common.

Bronchodilators can improve symptoms of breathlessness in patients with COPD [28], but are unlikely to be prescribed in the absence of a diagnosis. There may be many patients with heart failure and undiagnosed COPD who are not receiving potentially symptom-relieving treatment.

An alternative explanation for the prevalence of COPD in our cohort is that heart failure may act as a mimic of COPD [5], possibly due to alveolar and pulmonary interstitial oedema compressing small airways [25, 29]. Medical treatment of heart failure can correct abnormal spirometry in approximately 50% of patients with heart failure and ‘COPD’ after 6 months [6], and optimising heart failure medications and diuretic dose according to invasively measured pulmonary artery pressure can reduce the risk of admission with respiratory illness [30]. As some of our cohort were new referrals and naïve to treatment, it is possible that a proportion of patients with “heart failure and COPD” merely had more severe heart failure rather than concurrent pulmonary disease. However, it is important to note, regardless of whether obstructive spirometry (FEV_1_:FVC < 0.7) represents ‘true COPD’ or just more severe heart failure, it had no effect on mortality.

### The effect of a heart failure diagnosis on COPD

We found that a co-diagnosis of COPD and heart failure is far worse than having COPD alone. Many patients with heart failure have obstructive spirometry but the opposite may also be true: symptoms of heart failure such as breathlessness and peripheral oedema may be misinterpreted as COPD [31, 32], particularly if the diagnosis of COPD is established: there may be cases of undiagnosed heart failure amongst patients with COPD [33]. This is a much more important situation than missing COPD in a patient with heart failure given the potent effects of medical therapy for patients with HeFREF [1].

Cardiovascular death is common amongst patients with COPD [34–39), and beta-blockers might reduce the risk of mortality amongst patients with COPD [39]. Whether other treatments that target cardiovascular risk (such as spironolactone) can improve outcome in patients with COPD (and not heart failure) is unknown. Screening patients with COPD for heart failure using natriuretic peptides could identify those at greater risk of adverse outcome, and should be considered if there is any doubt that a patient’s symptoms are due to COPD alone.

### Study limitations

The limitations of retrospective analyses apply to our study and confounding factors cannot be excluded. Our data represent a snapshot of a single time point and no conclusions can be drawn on the importance of changing spirometry or the effect of heart failure treatment on spirometry amongst patients with heart failure over time. Our data did not include reversibility studies as part of the spirometry, nor did we have data on hospitalisation rates.

Patients in whom COPD was the obvious cause of their symptoms are unlikely to have been referred to a heart failure service. The patients in our study with COPD alone are likely to be those with relatively mild disease and might thus not be truly representative of the ‘real-world’ COPD population. The role of our clinic is to identify and treat patients with heart failure, not identifying the specific underlying cause of breathlessness. For that reason we are unable to comment on the cause of breathlessness in the sub-group of patients with neither heart failure nor COPD (*N* = 886), although it is interesting to note that the average body mass index of patients in this group was higher than that of patients with heart failure and/or COPD (Table 1) and meets the National Institute of Clinical Excellence (NICE) diagnostic criteria for obesity [40].

## Conclusions

The symptoms of heart failure and COPD overlap. Both carry an adverse prognosis. In patients with COPD, a coincident diagnosis of heart failure greatly worsens prognosis, but an additional diagnosis of COPD in patients with heart failure does not affect outcome.

## Electronic supplementary material

Below is the link to the electronic supplementary material.

**Supplementary figure 1**: CONSORT diagram. Abbreviations used: *COPD* chronic obstructive pulmonary disease; *HeFNEF* heart failure with a normal ejection fraction; *HeFREF* heart failure with a reduced ejection fraction (TIF 143 KB)

**Supplementary figure 2** Univariable correlation matrix for FEV_1_:FVC and other selected variables. All *P* values <0.005 unless otherwise stated, boxes shaded in grey represent non-significant correlations. Abbreviations used: *BMI* body mass index; *eGFR* estimated glomerular filtration rate; *FEV*_*1*_ forced expiratory volume in 1 second; *FVC* forced vital capacity; *NTproBNP* N-terminal prohormone of B-type natriuretic peptide; *NS* non-significant (TIF 218 KB)

Supplementary material 3 (DOC 120 KB)

Supplementary material 4 (DOC 117 KB)
