# Supplementary figures and images for: The impact of heart failure and chronic obstructive pulmonary disease on mortality in patients presenting with breathlessness
Source: Clin Res Cardiol. 2018 Aug 8;108(2):185–93. doi: 10.1007/s00392-018-1342-z (PMC6510798; doi:10.1007/s00392-018-1342-z)

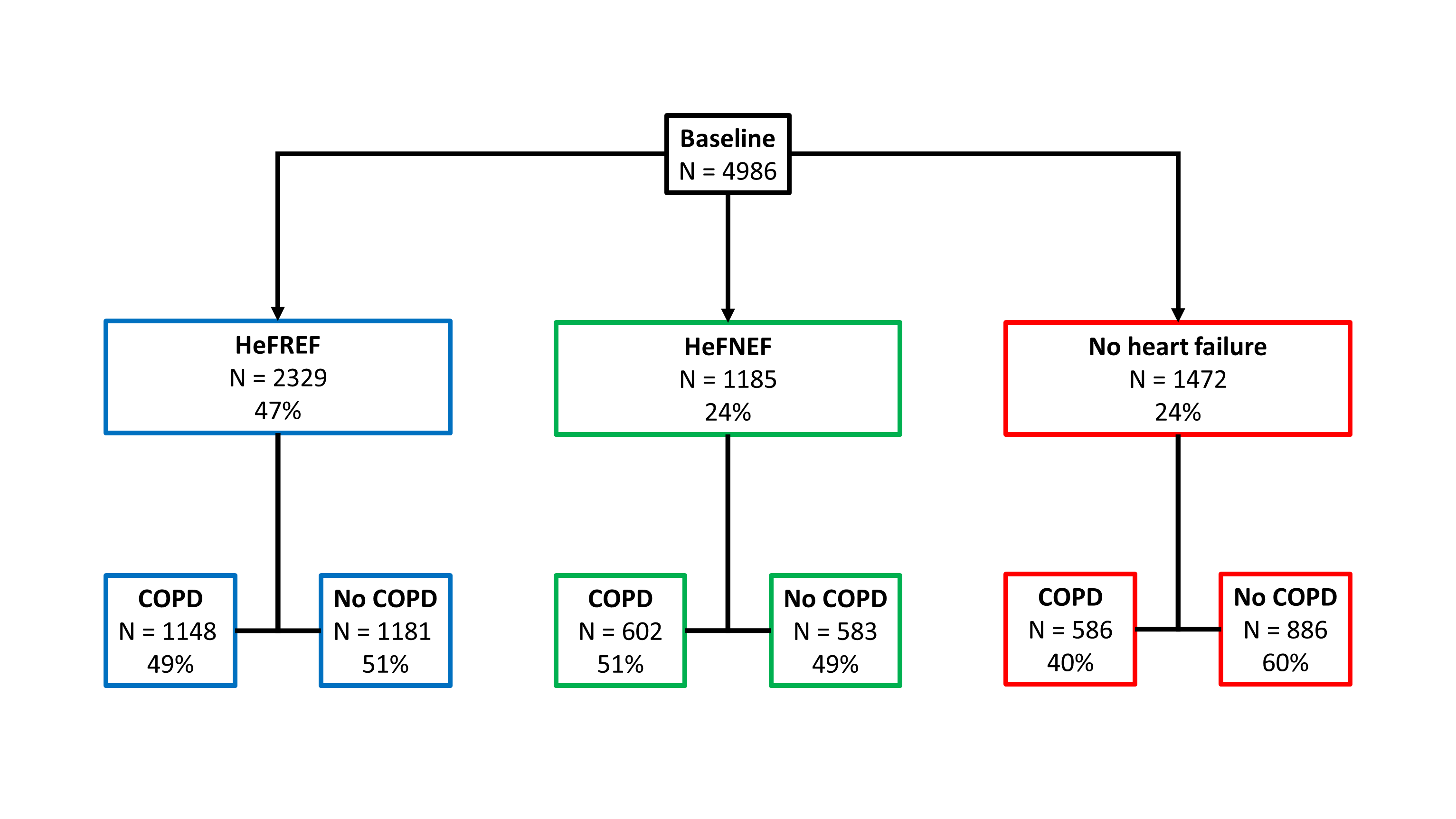

Supplement: Supplementary file 1 — Supplementary figure 1: CONSORT diagram. Abbreviations used: COPD chronic obstructive pulmonary disease; HeFNEF heart failure with a normal ejection fraction; HeFREF heart failure with a reduced ejection fraction (TIF 143 KB) [file 392_2018_1342_MOESM1_ESM.tif]

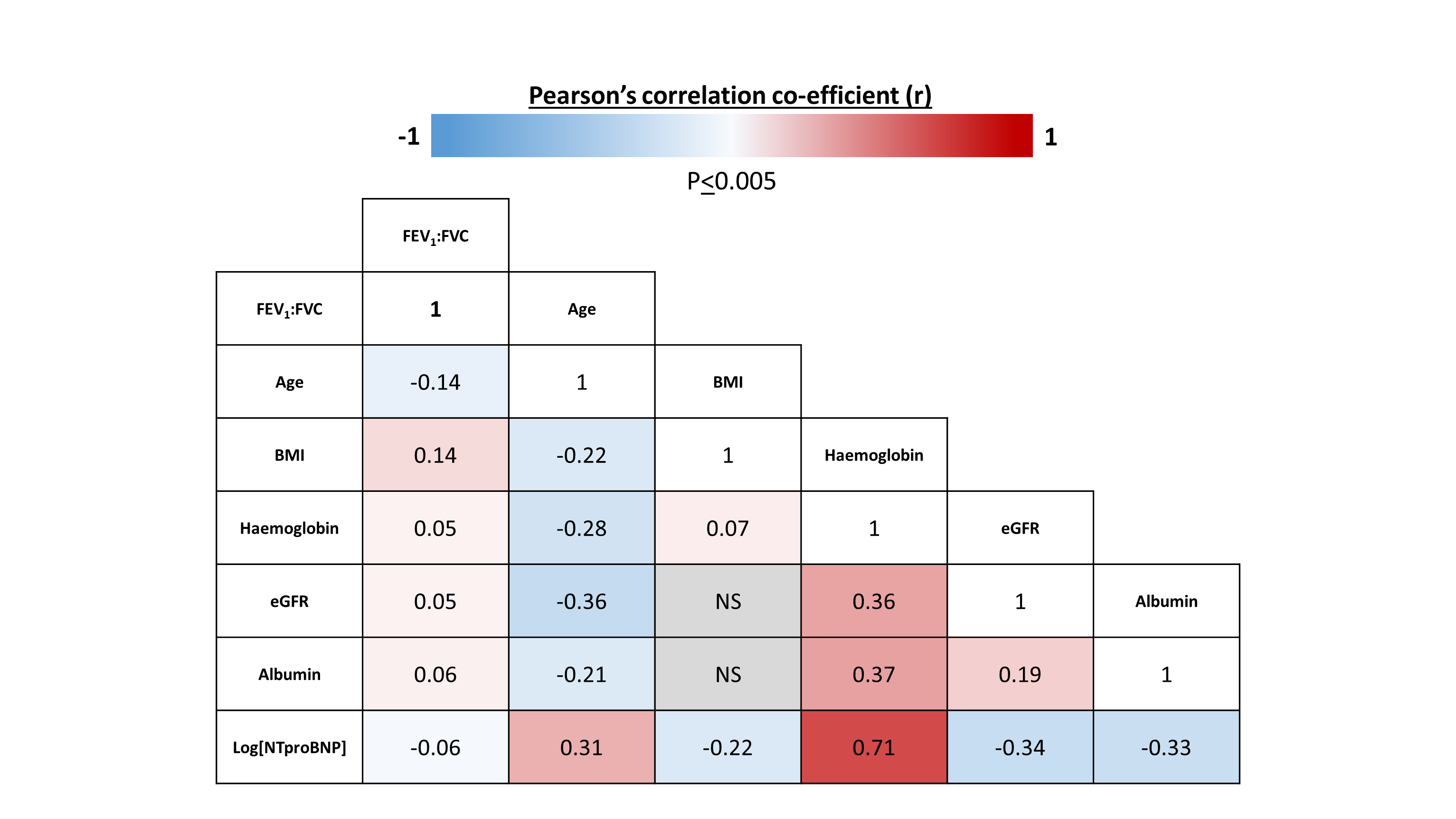

Supplement: Supplementary file 2 — Supplementary figure 2 Univariable correlation matrix for FEV1:FVC and other selected variables. All P values <0.005 unless otherwise stated, boxes shaded in grey represent non-significant correlations. Abbreviations used: BMI body mass index; eGFR estimated glomerular filtration rate; FEV1 forced expiratory volume in 1 second; FVC forced vital capacity; NTproBNP N-terminal prohormone of B-type natriuretic peptide; NS non-significant (TIF 218 KB) [file 392_2018_1342_MOESM2_ESM.tif]
