# Supplementary material for: The impact of heart failure and chronic obstructive pulmonary disease on mortality in patients presenting with breathlessness
Source: Clin Res Cardiol. 2018 Aug 8;108(2):185–93. doi: 10.1007/s00392-018-1342-z (PMC6510798; doi:10.1007/s00392-018-1342-z)
Supplement: Supplementary file 3 — Supplementary material 3 (DOC 120 KB) [file 392_2018_1342_MOESM3_ESM.doc]

| **Variable** | **Univariable** | | | **Multivariable** | | |
| --- | --- | --- | --- | --- | --- | --- |
|  | HR | X^2^ - Wald | P | HR | X^2^ - Wald | P |
| **Demographics** | | | | | | |
| **Age – years** | 1.06 (1.05 – 1.06) | 400 | <0.001 | 1.03 (1.02 – 1.04) | 20 | <0.001 |
| **Sex (male vs female)** | 1.22 (1.10 – 1.36) | 14 | <0.001 |  |  |  |
| **BMI – kg/m^2^** | 0.97 (0.96 – 0.98) | 54 | <0.001 | 0.98 (0.96 – 0.99) | 7 | 0.01 |
| **SR (vs not sinus rhythm)** | 1.74 (1.56 – 1.93) | 105 | <0.001 |  |  |  |
| **Diabetes (vs non-diabetic)** | 1.38 (1.22 – 1.55) | 27 | <0.001 |  |  |  |
| **IHD (vs no IHD)** | 1.46 (1.32 – 1.61) | 54 | <0.001 | 1.30 (1.05 – 1.61) | 6 | 0.02 |
| **Current or ex-smoker (vs never-smoker)** | 1.42 (1.26 – 1.60) | 34 | <0.001 | 1.44 (1.16 – 1.78) | 11 | 0.001 |
| **Symptoms** | | | | | | |
| **NYHA Class (III/IV vs I/II)** | 2.33 (2.11 – 2.58) | 264 | <0.001 | 1.56 (1.28 – 1.91) | 19 | <0.001 |
| **Blood results** | | | | | | |
| **Log[NTproBNP] – ng/L** | 3.24 (2.98 – 3.53) | 719 | <0.001 | 1.87 (1.52 – 2.32) | 34 | <0.001 |
| **Haemoglobin – g/dL** | 0.77 (0.75 – 0.80) | 299 | <0.001 |  |  |  |
| **Sodium – mmol/L** | 0.92 (0.91 – 0.93) | 125 | <0.001 |  |  |  |
| **Potassium – mmol/L** | 1.04 (0.94 – 1.16) | <1 | 0.43 | ― | ― | ― |
| **Chloride – mmol/L** | 0.92 (0.91 – 0.93) | 226 | <0.001 |  |  |  |
| **Bicarbonate – mmol/L** | 1.01 (0.99 – 1.03) | <1 | 0.41 | ― | ― | ― |
| **eGFR – ml/min/1.73m^2^** | 0.97 (0.97 – 0.98) | 456 | <0.001 |  |  |  |
| **Albumin – g/l** | 0.86 (0.85 – 0.87) | 557 | <0.001 | 0.94 (0.91 – 0.96) | 22 | <0.001 |
| **Spirometry** | | | | | | |
| **FEV_1_:FVC** | 0.40 (0.30 – 0.54) | 36 | <0.001 |  |  |  |
| **FEV_1_:FVC <0.7 (vs ≥0.7)** | 1.43 (1.29 – 1.58) | 49 | <0.001 |  |  |  |
| **Medications** | | | | | | |
| **Loop diuretic (vs no loop diuretic)** | 2.92 (2.59 – 3.30) | 301 | <0.001 | 1.44 (1.14 – 1.82) | 9 | 0.003 |
| **ACEI/ARB (vs no ACEI/ARB)** | 0.80 (0.72 – 0.90) | 16 | <0.001 |  |  |  |
| **Beta-blocker (vs no beta-blocker)** | 0.88 (0.80 – 0.98) | 6 | 0.016 | 0.82 (0.68 – 0.99) | 4 | 0.04 |
| **MRA (vs no MRA)** | 0.62 (0.55 – 0.70) | 63 | <0.001 |  |  |  |
| **Echocardiography** | | | | | | |
| **Severe LVSD (vs not severe)** | 1.76 (1.57 – 1.98) | 92 | <0.001 |  |  |  |
| **LAD – cm** | 1.45 (1.36 – 1.54) | 129 | <0.001 |  |  |  |
| **LVEF by Simpsons – %** | 0.98 (0.97 – 0.98) | 117 | <0.001 |  |  |  |
| **HeFREF vs HeFNEF** | 1.02 (0.91 – 1.14) | <1 | 0.73 | ― | ― | ― |

**Supplementary table 1 – Univariable and multivariable Cox regression analysis for variables associated with all-cause mortality during 5-year follow up for all patients.**

**Legend**

Only variables associated with outcome in univariable analysis (p<0.05) were entered in multivariable models. Variables with >10% missing values were excluded. Non-significant variables in multivariable analysis are not presented. FEV_1_:FVC as a continuous variable and FEV_1_:FVC <0.7 as a categorical variable were entered separately into the model. Variables included in the multivariable model included: age, sex, BMI, presence of diabetes, presence of IHD, smoking status, NYHA class, log[NTproBNP], haemoglobin, sodium, chloride, eGFR, albumin, FEV_1_:FVC, FEV_1_:FVC <0.7, loop diuretic status, ACEI or ARB status, beta-blocker status, MRA status, and presence of severe LVSD.

List of abbreviations used: N – number; BMI – body mass index; SR – sinus rhythm; IHD – ischaemic heart disease; NYHA – New York Heart Association; NTproBNP – N-terminal B-type natriuretic peptide; eGFR – estimated glomerular filtration rate; FEV_1_ – forced expiratory volume in one second; FVC – forced vital capacity; MRA – mineralocorticoid receptor antagonist; ACEI – angiotensin converting enzyme inhibitor; ARB – angiotensin receptor blocker; βB – beta-blocker; LVSD – left ventricular systolic dysfunction; LAD – left atrial diameter; LVEF – left ventricular ejection fraction; HeFREF – heart failure with reduced ejection fraction; HeFNEF – heart failure with normal ejection fraction.
