# Supplementary material for: The impact of heart failure and chronic obstructive pulmonary disease on mortality in patients presenting with breathlessness
Source: Clin Res Cardiol. 2018 Aug 8;108(2):185–93. doi: 10.1007/s00392-018-1342-z (PMC6510798; doi:10.1007/s00392-018-1342-z)
Supplement: Supplementary file 4 — Supplementary material 4 (DOC 117 KB) [file 392_2018_1342_MOESM4_ESM.doc]

| **Variable** | **Univariable** | | | **Multivariable** | | |
| --- | --- | --- | --- | --- | --- | --- |
|  | HR | X^2^ - Wald | P | HR | X^2^ - Wald | P |
| **Demographics** | | | | | | |
| **Age – years** | 1.05 (1.04 – 1.05) | 232 | <0.001 | 1.03 (1.02 – 1.04) | 19 | <0.001 |
| **Sex (male vs female)** | 1.04 (0.92 – 1.16) | <1 | 0.55 |  |  |  |
| **BMI – kg/m^2^** | 0.97 (0.96 – 0.98) | 36 | <0.001 |  |  |  |
| **SR (vs not sinus rhythm)** | 0.85 (0.76 – 0.95) | 8 | 0.005 |  |  |  |
| **Diabetes (vs non-diabetic)** | 1.25 (1.10 – 1.42) | 11 | 0.001 |  |  |  |
| **IHD (vs no IHD)** | 1.20 (1.08 – 1.34) | 11 | 0.001 | 1.31 (1.06 – 1.63) | 6 | 0.01 |
| **Current or ex-smoker (vs never-smoker)** | 1.23 (1.08 – 1.40) | 10 | 0.002 | 1.51 (1.19 – 1.91) | 12 | 0.001 |
| **Symptoms** | | | | | | |
| **NYHA Class (III/IV vs I/II)** | 1.97 (1.76 – 2.19) | 148 | <0.001 | 1.51 (1.21 - 1.87) | 14 | <0.001 |
| **Blood results** | | | | | | |
| **Log[NTproBNP] – ng/L** | 3.27 (2.90 – 3.68) | 370 | <0.001 | 1.92 (1.49 – 2.47) | 26 | <0.001 |
| **Haemoglobin – g/dL** | 0.80 (0.78 – 0.83) | 190 | <0.001 |  |  |  |
| **Sodium – mmol/L** | 0.94 (0.93 – 0.96) | 58 | <0.001 |  |  |  |
| **Potassium – mmol/L** | 0.98 (0.88 – 1.10) | <1 | 0.73 |  |  |  |
| **Chloride – mmol/L** | 0.94 (0.93 – 0.95) | 109 | <0.001 |  |  |  |
| **Bicarbonate – mmol/L** | 1.00 (0.98 – 1.02) | <1 | 0.96 |  |  |  |
| **eGFR – ml/min/1.73m^2^** | 0.98 (0.98 – 0.98) | 246 | <0.001 |  |  |  |
| **Albumin – g/l** | 0.88 (0.87 – 0.90) | 340 | <0.001 | 0.93 (0.90 – 0.96) | 24 | <0.001 |
| **Spirometry** | | | | | | |
| **FEV_1_:FVC** | 0.52 (0.38 – 0.73) | 15 | <0.001 |  |  |  |
| **FEV_1_:FVC <0.7 (vs ≥0.7)** | 1.24 (1.11 – 1.38) | 15 | <0.001 |  |  |  |
| **Medications** | | | | | | |
| **Loop diuretic (vs no loop diuretic)** | 2.05 (1.79 – 2.36) | 102 | <0.001 | 1.32 (1.02 – 1.71) | 5 | 0.04 |
| **ACEI/ARB (vs no ACEI/ARB)** | 0.86 (0.76 – 0.97) | 7 | 0.01 |  |  |  |
| **Beta-blocker (vs no beta-blocker)** | 0.71 (0.64 – 0.80) | 38 | <0.001 |  |  |  |
| **MRA (vs no MRA)** | 1.23 (1.09 – 1.40) | 11 | 0.001 |  |  |  |
| **Echocardiography** | | | | | | |
| **Severe LVSD (vs not severe)** | 1.23 (1.09 – 1.38) | 11 | 0.001 |  |  |  |
| **LAD – cm** | 1.20 (1.12 – 1.29) | 25 | <0.001 |  |  |  |
| **LVEF by Simpsons – %** | 1.00 (0.99 – 1.00) | 3 | 0.07 |  |  |  |
| **HFrEF vs HFnEF** | 1.02 (0.91 – 1.14) | <1 | 0.73 | ― | ― | ― |

**Supplementary table 2 – Univariable and multivariable Cox regression analysis for variables associated with all-cause mortality during 5 year follow up for all patients with heart failure (N=3515).**

**Legend**

Only variables associated with outcome in univariable analysis (p<0.05) were entered in multivariable models. Variables with >10% missing values were excluded. Non-significant variables in multivariable analysis are not presented. FEV_1_:FVC as a continuous variable and FEV_1_:FVC <0.7 as a categorical variable were entered separately into the model. Variables included in the multivariable model included: age, BMI, presence of diabetes, presence of IHD, smoking status, NYHA class, log[NTproBNP], haemoglobin, sodium, chloride, eGFR, albumin, FEV_1_:FVC, FEV_1_:FVC <0.7, loop diuretic status, ACEI or ARB status, beta-blocker status, MRA status, and the presence of severe LVSD.

List of abbreviations used: N – number; BMI – body mass index; SR – sinus rhythm; IHD – ischaemic heart disease; NYHA – New York Heart Association; NTproBNP – N-terminal B-type natriuretic peptide; eGFR – estimated glomerular filtration rate; FEV_1_ – forced expiratory volume in one second; FVC – forced vital capacity; MRA – mineralocorticoid receptor antagonist; ACEI – angiotensin converting enzyme inhibitor; ARB – angiotensin receptor blocker; βB – beta-blocker; LVSD – left ventricular systolic dysfunction; LAD – left atrial diameter; LVEF – left ventricular ejection fraction; HeFREF – heart failure with reduced ejection fraction; HeFNEF – heart failure with normal ejection fraction.
